# Neighbourhood-level age-friendliness in urban environments: associations with functional independence and physical activity in older adults after a fall

**DOI:** 10.3389/fpubh.2026.1839024

**Published:** 2026-05-29

**Authors:** Tim Stuckenschneider, Frank Oswald, Adele Grenz, Laura Himmelmann, Nina Marie Schmidt, Elisa-Marie Speckmann, Tania Zieschang, Kathrin Boerner

**Affiliations:** 1 Department for Health Services Research, Geriatric Medicine/School VI- School of Medicine and Health Services, Carl von Ossietzky Universität Oldenburg, Oldenburg, Lower Saxony, Germany; 2 Interdisciplinary Ageing Research (IAW), Goethe University Frankfurt, Frankfurt, Germany; 3 Prevention and Rehabilitation Research/ School VI- School of Medicine and Health Services, Carl von Ossietzky Universität Oldenburg, Oldenburg, Lower Saxony, Germany

**Keywords:** AFCCQ, autonomy, emergency department, environmental gerontology, life-space mobility, older adults

## Abstract

**Background:**

Population ageing and urbanisation are reshaping living environments and raising new challenges for promoting health and independence in later life. Age-friendly city initiatives aim to support active ageing, yet the relationship between environmental conditions, housing factors, and functional outcomes remains insufficiently understood.

**Methods:**

This cross-sectional study analysed data from participants of the SeFallED study in Oldenburg, Germany, which enrolled community-dwelling older adults (≥60 years) presenting to the emergency department due to a fall. Physical activity was assessed using accelerometry and self-report, life-space mobility using the Life-Space Assessment, and independence using instrumental activities of daily living (iADL). Neighbourhood-level age-friendliness was quantified using the Age-Friendly Cities and Communities Questionnaire (AFCCQ). Multiple linear regression models examined associations between environmental factors, housing characteristics, and outcomes.

**Results:**

In total, 179 participants (mean age 77.3 ± 7.9 years) were included in the study. Neighbourhood-level age-friendliness showed limited associations with active physical activity outcomes, but was associated with sedentary behaviour. Higher age and widowhood were consistently linked to lower activity levels, while higher age-friendliness scores were significantly associated with lower life-space mobility and lower independence, indicating greater functional limitations.

**Conclusion:**

Findings suggest that age-friendly environments may be particularly relevant for older adults after a fall with reduced mobility and independence. Evaluations of age-friendly city initiatives should, therefore, consider not only behavioural activation but also the maintenance of functioning in vulnerable populations. Longitudinal research is needed to clarify causal pathways and the dynamic interplay between person and environment in ageing societies.

## 1 Introduction

Urbanisation and demographic change are transforming living conditions worldwide. By 2050, an estimated 68.4% of the global population will reside in urban areas (1). In Germany, approximately 40.5% of the population currently lives in cities and metropolitan regions (1), while at the same time the population is steadily aging. Projections suggest that by 2050, the number of adults aged 80 years and older will nearly triple (2). These demographic shifts are increasingly shaping urban life and pose new challenges for urban development and municipal planning.

A central aspect of these challenges concerns housing. Remaining in one’s private home represents not only the reality for most older adults but also their strong personal preference (3). In Germany, around 96% of people over 65 years and about 89% of those over 80 still live in private homes, and nearly half of the latter group maintains independence without recourse to formal care services (4). Preserving independence is a primary goal for older adults, which has far-reaching implications for late-life wellbeing. Mentally and physically active ageing, established key determinants of health, social participation, and quality of life (5) support independence.

From the perspective of environmental gerontology, mobility and physical activity are not solely determined by individual competences but are embedded in specific socio-spatial contexts. They can arise from continuous interaction between the individual and the environment (6, 7). More recent person-environment (P-E) exchange models further distinguish between goal-oriented processes of ‘agency’—such as mobility and physical activity within the everyday home and neighbourhood environment—and processes of ‘belonging’, including the perceived meaningful connection to one’s home or perceived age-friendliness of the environment, which carry substantial cognitive and emotional value (7–9). Both types of processes are considered important determinants of broader outcomes, such as health or wellbeing (7–9). In the context of the present study, we focus on the interplay of these processes, assuming that physical activity, life-space mobility as well as daily independence are associated with perceived age-friendliness of the urban neighbourhood. Accordingly, the residential environment may influence these outcomes in later life, thereby offering potential targets for intervention.

Furthermore, household composition, such as living alone or with a partner, have also been shown to influence physical activity (10–12), highlighting an additional perspective potentially influencing independence and participation in older age. However, the interplay between personal and environmental factors, and the extent to which they shape different dimensions of physical activity in older adults, remain insufficiently understood. One reason for this knowledge gap lies in the limited availability of standardized instruments to assess structural conditions in cities.

To address this issue, the World Health Organization (WHO) launched the “Age-friendly Cities and Communities” (AFCC) initiative, with the aim of enabling active and independent ageing and fostering wellbeing through social and economic participation (13). The associated “Age-friendly Cities and Communities Questionnaire” (AFCCQ) (14), validated in German in 2024 (15), provides an important tool for assessing relevant environmental conditions. Previous studies have emphasized the critical role of housing quality and objective living conditions (e.g., accessibility, barrier-free design, orientation) as well as neighbourhood and community-level factors, at both national and international levels (16). The WHO’s AFCC framework similarly emphasises housing and neighbourhood environments as key determinants of participation and independence, and these considerations have also shaped national policy strategies (17, 18).

Against this background, the present study investigates how personal housing factors (e.g., living alone or with a partner) and urban environmental factors influence physical activity and life-space mobility among older adults in a medium-sized German city. By identifying the interplay between these factors, we aim to generate insights into the relevance of environmental and personal factors, which may inform the design of age-friendly cities that promote activity, participation, and independence in later life.

## 2 Methods

### 2.1 Sample

Participants included in the analysis were taken from the Sentinel Fall Presenting to the Emergency Department (SeFallED) study, conducted at the Carl von Ossietzky University Oldenburg, Germany. The primary aim of SeFallED is to investigate long-term functional trajectories of older adults presenting to the ED following a fall that did not result in hospital admission. The study protocol has been described in detail previously (19). The study was prospectively registered in the German Clinical Trial Register (DRKS-ID: 00025949), approved by the Medical Ethics Committee of the University of Oldenburg (number 2021–120) and conducted in accordance with the Declaration of Helsinki.

Participants were recruited between November 2021 and January 2023. Inclusion criteria were (1) age ≥60 years, (2) presentation to the ED of the Klinikum Oldenburg or Evangelisches Krankenhaus Oldenburg after a fall and discharge within 72 h and (3) informed consent. Exclusion criteria included: (1) life expectancy less than 3 months, (2) unstable medical, neurological or psychiatric condition, (3) bedridden or being unable to walk without support of another person, (4) residence more than 40 km away from the research centre, (5) acute psychosis or social aggression, (6) inability to communicate verbally in German or English. For the present sub-study, additional exclusion criteria were applied: (1) institutionalization, (2) current employment (i.e., not yet retired) and (3) residence outside the city of Oldenburg.

### 2.2 Data collection

#### 2.2.1 Housing situation

Participant characteristics included age in years and sex. Personal housing characteristics and living arrangements were addressed as follows. Housing type (non-barrier-free apartment, non-barrier-free house, barrier-free housing) and living situation (living alone, living with a partner, widowed) were entered into the models using dummy coding, with non-barrier-free apartment and living alone serving as reference categories. Sex was coded as male and female. Age was included as a continuous variable.

#### 2.2.2 Physical activity

Physical activity was measured using a three-axial accelerometer (activPAL4™, PAL Technologies Ltd., Glasgow, UK) (20), which was attached to the participants’ thigh by a trained study team member. An additional consent form was obtained for the collection of sensor data. If participants provided their consent, they were instructed to wear the sensor for seven consecutive days. A minimum of 24 h of data collection for at least five consecutive days was required for inclusion of the data in the analysis, as recommended by previous literature (21). Number of steps per day, one parameter derived from the wearable device, has been validated as a proxy for measuring physical activity in community-dwelling older adults (22). Furthermore, time spent sedentary, which negatively impacts health, was obtained from the sensor. Additionally, self-reported physical activity was assessed using the German-PAQ-50+, which is a well-established questionnaire in adults over the age of 50 years and a feasible measure of self-reported physical activity (23). Total time spent on physical activity (in minutes) as well as energy spent in these activities (total kcal) was used for further analysis.

#### 2.2.3 Life-space mobility

Mobility range was assessed with the German Life-space Assessment (LSA-D), which rates mobility frequency across five life-space levels (indoors, immediate surroundings, local neighbourhood, within town, beyond town) during the preceding 4 weeks, and whether assistant devices or personal help was needed (24). The total score was used, with higher values indicating greater life-space mobility and autonomy.

#### 2.2.4 Daily independence

Instrumental activities of daily living (iADL) were assessed using the Lawton and Brody iADL scale. This scale consists of eight items evaluating an individual’s ability to perform the following activities: using a telephone, shopping, meal preparation, housekeeping, laundry, transportation, medication management, and financial management. Each item is scored as 0 (lower ability) or 1 (higher ability), yielding a total score ranging from 0 to 8, with higher scores indicating greater functional independence (25).

#### 2.2.5 Age-friendliness of the neighbourhood

Results from the recent validation of the Age-Friendly Cities and Communities Questionnaire (AFCCQ-DE) in a sample of 905 older adults in Oldenburg were also incorporated (15). Data from this validation study were linked to participants of the SeFallED study. For this purpose, the residential addresses of the included participants were used to assign neighbourhood-level AFCCQ scores, based on the mean values calculated for the corresponding city districts in the validation study (26). Each participant was assigned an overall AFCCQ score as well as scores for the nine subdimensions of the questionnaire: housing; social participation; respect and social inclusion; civic participation and employment; communication and information; community support and health services; outdoor spaces and buildings; transportation; and financial situation. As city districts tend to comprise relatively homogeneous populations, these neighbourhood-level data were considered representative and were, therefore, applied to the individual study sample. In addition to the overall score, the subscales were aggregated into three domains, which were physical environment, social environment, and community-based services, to allow a more differentiated analysis of their respective influences (27).

### 2.3 Data analysis

Statistical analyses were conducted using IBM SPSS Statistics (Version 29). Statistical significance was set at *α* = 0.05. Associations between neighbourhood-level AFCCQ and multiple dimensions of physical activity, sedentary behaviour, life-space mobility, and iADLs were examined using generalized estimating equation (with robust standard errors). This approach accounts for potential non-independence of observations by clustering participants within city district. Initial multilevel linear models with random intercepts for city districts were estimated but did not converge, indicating negligible between-area variance. Therefore, GEE models were used as a robust alternative.

Personal housing characteristics and living arrangements were included as covariates. For each dependent variable, four separate models were specified. Model 1 included the AFCCQ total score as the primary predictor, adjusted for age, sex, housing type (dummy variables), and living situation (dummy variables). Models 2, 3 and 4 examined domain-specific associations by replacing AFCCQ total score with one AFCCQ subdomain score per model (physical environment, social environment, and community-based services), while retaining the same covariates. Robust standard errors were used to account for clustering. Multicollinearity was assessed using variance inflation factors (VIF) and tolerance values, all of which were within acceptable ranges.

## 3 Results

A total of 179 participants fulfilled the inclusion criteria for this sub study of the SeFallED project and were included in this sub analysis. Approximately two thirds of the participants were female. Detailed demographic and baseline characteristics are presented in Table 1.

**Table 1:** Participant characteristics.

*n* = 179		Mean ± (SD)	Minimum	Maximum
Age (years)		77.3 ± 7.9	60	93
Sex (female/male; *n*)		115/64		
Body Mass Index (BMI)		27.4 ± 5.4	16.5	46.4
Daily Independence (iADL)		7.2 ± 1.7	1	8
Self-reported PhysicalActivity (PA) (*n* = 173)	Minutes	81.2 ± 72.1	0	461
	Kcal	6250.83 ± 5846.6	0	34919.64
Sensor-based PhysicalActivity (PA) (*n* = 136)	Step Count	5927.1 ± 3435.4	149	16,785
	Sedentary Time	652.6 ± 152.7	327	1,203
Age-friendliness (AFCCQ total and subscores)	Total Score	18.4 ± 2.0	15	21.9
	Physical environment	2.1 ± 0.2	1.7	2.7
	Social environment	2.2 ± 0.2	1.7	2.7
	Community-based services	2.2 ± 0.3	1.7	2.8
Life-space Mobility (*n* = 170)		63.5 ± 27.8	4	120

### 3.1 Physical activity

Across most models capturing physical activity, neighbourhood-level age-friendliness (AFCCQ total score and domain scores) was not significantly associated with self-reported activity level (in minutes), energy expenditure (kcal), or sensor-based step count. Statistically significant associations are listed in Table 2 and full analysis details in the Supplementary material.

**Table 2:** Associations of neighbourhood-level age-friendliness (AFCCQ) with physical activity.

Predictor	B (SE)	95% CI	*p*-value
Physical activity (activity level, minutes)			
Age (years)	−1.88 (0.45)	−2.76 to −1.00	<0.001
Housing (non-barrier-free)	21.10 (8.43)	4.58 to 37.63	0.012
Widowed	−43.03 (14.36)	−71.18 to −14.89	0.003
Social environment	−30.99 (13.81)	−58.04 to −3.93	0.025
Physical activity (energy expenditure, kcal)			
Sex (female vs. male)	−2409.66 (743.68)	−3867.25 to −952.06	0.001
Age (years)	−163.56 (43.35)	−248.52 to −78.60	<0.001
Housing: non-barrier-free	1586.43 (622.67)	366.01 to 2806.84	0.011
Widowed	−2983.67 (1110.41)	−5160.03 to −807.32	0.007
Physical activity (steps per day)			
Age (years)	−160.27 (36.34)	−231.50 to −89.03	<0.001
Sedentary time (minutes)			
AFCCQ total score	9.87 (3.23)	3.53 to 16.21	0.002
Age (years)	2.38 (0.84)	0.73 to 4.03	0.005
Physical environment	137.01 (26.26)	85.53 to 188.49	<0.001

For activity level, the social environment domain was significantly associated with activity level, indicating lower activity levels among individuals living in city districts with higher social environment age-friendliness scores. No significant associations were found for AFCCQ total score or the other two subdomains. Across all models, increasing age was significantly associated with lower activity levels, and being widowed was associated with lower activity. In addition, living in a non-barrier-free house was associated with higher activity levels compared with living in a barrier-free house (detailed results are provided in Supplementary Table S2).

For self-reported energy expenditure in kcal, AFCCQ total score and domain scores were not significantly associated with kcal expenditure. Age, female sex, and being widowed were significantly associated with lower kcal expenditure. Living in a non-barrier-free house was associated with higher kcal expenditure (Supplementary Table S2).

For step count, neither AFCCQ total nor domain scores showed significant associations. Only age showed a consistent significant negative association across models (Supplementary Table S2).

Higher AFCCQ total scores and higher physical environment age-friendliness scores were significantly associated with greater sedentary time. Increasing age was also significantly associated with greater sedentary across all models (please see Supplementary Table S2).

### 3.2 Life-space mobility

Statistically significant associations are listed in Table 3 and full analysis details in Supplementary Table S3. The social environment domain was significantly negatively associated with life-space mobility, indicating lower life-space mobility among individuals living in areas with higher perceived social environment age-friendliness. Increasing age and being widowed were consistently associated with lower life-space mobility across all models. No further significant associations were revealed.

**Table 3:** Associations of neighbourhood-level age-friendliness (social environment) with life-space mobility.

Predictor	B (SE)	95% CI	*p*-value
Life-space mobility			
Social environment	−16.10 (5.88)	−27.63 to −4.58	0.006
Age (years)	−1.44 (0.16)	−1.76 to −1.13	<0.001
Widowed	−10.85 (4.60)	−19.86 to −1.84	0.018

### 3.3 Daily independence

Statistically significant associations with daily independence in terms of iADL are listed in Table 4 and full analysis details in Supplementary Table S4. For iADL, neighbourhood-level age-friendliness showed consistent associations. Higher AFCCQ total scores were significantly associated with lower iADL scores, indicating greater dependence. Similar associations were observed for the social environment age-friendliness. Increasing age, living in barrier-free housing as well as being widowed were also associated with lower iADL scores.

**Table 4:** Associations of neighbourhood-level age-friendliness (AFCCQ) with instrumental activities of daily living (iADLs).

Predictor	B (SE)	95% CI	*p*-value
Instrumental activities of daily living			
AFCCQ total score	−0.18 (0.03)	−0.24 to −0.11	<0.001
Age (years)	−0.08 (0.02)	−0.12 to −0.03	0.001
Housing: barrier-free	−0.70 (0.30)	−1.29 to −0.12	0.018
Living with partner	−0.39 (0.14)	−0.67 to −0.12	0.005
Widowed	−0.78 (0.19)	−1.15 to −0.41	<0.001
Social environment	−1.03 (0.21)	−1.44 to −0.62	<0.001
Community-based services	−1.29 (0.27)	−1.81 to −0.77	<0.001

## 4 Discussion

This study examined associations between neighbourhood-level age-friendliness, some personal housing factors, and different dimensions of physical activity, life-space mobility, and daily independence in terms of iADLs in older adults after a recent fall. Contrary to our initial expectation, age-friendliness was not associated with higher levels of physical activity or step counts. Instead, age-friendliness indicators were consistently related to life-space mobility and daily independence indicating functional outcomes in an unexpected direction. Higher neighbourhood-level age-friendliness, particularly regarding the social environment, was associated with lower life-space mobility and greater daily dependence. These results suggest that age-friendly neighbourhoods may be particularly relevant for older adults with existing mobility restrictions and functional limitations. This finding is consistent with previous research in very old community-dwelling adults living in urban areas, which shows that in advanced age and the presence of functional limitations, cognitively and emotionally meaningful bonding processes, such as neighbourhood level age-friendliness, play a protective role in wellbeing (28).

Reducing sedentary behaviour and increasing physical activity are important cornerstones, particularly in older adults, of the WHO’s global action plan on physical activity (29). Age-friendly city initiatives similarly aim to create physical and social environments that support active ageing (30). However, our results do not support the assumption that neighbourhood-level age-friendliness directly translates into higher levels of physical activity. Previous studies have demonstrated positive associations between neighbourhood walkability, safety and convenience and physical activity, particularly among middle-aged adults (31–33). In older populations, however, findings have been more heterogeneous. It has been argued that personal factors such as self-efficacy, health status, and functional capacity, may play a more prominent role in shaping physical activity behaviour in later life, underscoring the importance of multilevel approaches (34, 35).

The absence of positive associations between neighbourhood-level age-friendliness and active physical activity outcomes in our analyses may also be attributable to the relatively homogenous urban context. AFCCQ data from the validation study indicated general satisfaction with age-friendliness in Oldenburg (15), potentially limiting variability and reducing the likelihood of detecting associations with physical activity. In contrast previous studies and a systematic review comparing more diverse cities and neighbourhoods have reported associations between urban walkability and activity levels (36, 37). Future research should, therefore, incorporate samples from more heterogeneous urban settings and include individual-level psychological and health-related factors to better capture the dynamic interplay between environmental conditions and personal characteristics. Recruiting participants through settings such as emergency departments may facilitate access to more socioeconomically and culturally diverse populations (38, 39), as previous research suggests that emergency departments are disproportionately utilised by individuals from lower socioeconomic backgrounds (40).

Higher neighbourhood-level age-friendliness, particularly within the physical environment domain, was associated with greater sedentary time. This finding appears counterintuitive, as previous research has suggested that more age-friendly neighbourhoods are associated with lower sedentary time. For example, Asiamah et al. (41) reported in flatter, more age-friendly neighbourhoods compared to hilly environments, while Chang et al. (42) found that greater sidewalk availability was associated with fewer sedentary bouts. One possible explanation is that older adults with mobility limitations spend more time within their immediate residential environment and, therefore, place greater value on accessible and supportive neighbourhood structures. As all participants in the present study had recently experienced a fall, age-friendly neighbourhood features may also facilitate recovery and ageing-in-place through easier access to local support and services, potentially contributing to higher sedentary time. Future research in more heterogeneous populations and settings is needed to further clarify the relationship between sedentary behaviour and neighbourhood age-friendliness, particularly given the importance of reducing sedentary behaviour within the WHO’s global action plan on physical activity (29).

Increasing age was strongly associated with lower levels of physical activity across models, consistent with previous research and global trends as described by Strain et al. (43). These findings underscore the continued importance of creating environments and programs that promote physical activity across the entire later life course, with particular attention to the oldest-old population. In addition to age, widowhood was associated with lower physical activity. Although some studies suggest that physical activity may temporarily increase in men following spousal loss, such short-term changes often attenuate over time (44). Widowhood represents a major life transition that may involve psychological distress, social isolation, and disruption of daily routines, all of which may contribute to reduced activity levels (45). Evidence that partners influence each other’s health behaviours, including physical activity (46), further suggests that the loss of a spouse may destabilize established behavioural patterns. These findings highlight the importance of targeted strategies addressing older adults living alone following bereavement. Future research should more systematically examine living arrangements and dyadic influences to better understand how social contexts shape physical activity in later life.

Life-space mobility and daily independence showed associations with neighbourhood-level age-friendliness; however, these associations were observed in an unexpected negative direction. Higher age-friendliness, particularly regarding the social environment domain, was associated with lower life-space mobility and lower iADL scores, indicating reduced mobility and greater functional limitations. Previous research has often described higher physical activity levels and better functional status as drivers of social participation and engagement (47, 48). This pattern was not reflected in the present findings. One possible interpretation is that age-friendly environments may function more as supportive contexts rather than as drivers of physical activity or spatial mobility. The social environment domain appeared particularly relevant, suggesting that neighbourhood-level social support structures may be especially important for older adults with functional limitations. This interpretation is consistent with the perspective proposed by Bayat and colleagues, who suggested that a smaller life-space within a dense, age-friendly environment may represent an adaptive and contextually appropriate pattern rather than a negative outcome (49). However, it is also possible that participants with poorer functional status remain longer in more age-friendly environments or rely more strongly on their immediate surroundings and, thus, evaluate them more positively. Longitudinal studies are needed to disentangle these hypotheses and clarify potential causal and bidirectional relationships. From a municipal perspective, these findings highlight the potential importance of strengthening social participation opportunities and ensuring access to community-based services, including affordable programs and accessible transport options for older adults with mobility restrictions. Physical activity and mobility, in contrast, may be more strongly influenced by individual-level factors such as personality traits, educational background, social networks, and self-efficacy. Future research should include both older adults without care needs and those with higher care needs to better disentangle whether age-friendly environments primarily act as activators or compensatory supports. Incorporating measures of care need, social constellation, and personal resources may provide more precise guidance for policymakers and urban planners in ageing societies.

The present findings can be interpreted within the framework of P-E interaction models and conceptual critique (7), although environmental press theory should no longer be adopted in an overly simplified form and translated into applications. The conceptual ambiguity in classifying the everyday activities and aspects of mobility that we have addressed – and which are significant in the daily lives of older people – as well as their perceived age-friendliness and the resulting daily independence, can help to identify the differential relationships between these processes and the need for rigorous measurement of them. Within this perspective, the observed inverse associations between specific neighbourhood-level age-friendliness domains, life-space mobility and daily independence do not necessarily indicate detrimental environmental effects but can reflect long term buffering processes of P-E-belonging and agency for specific risk groups in very old age. Rather, they may reflect greater reliance on environmental support among older adults with reduced functional capacity. Although causal inferences cannot be drawn from this cross-sectional study, these findings suggest that age-friendly city initiatives should be evaluated not only by their potential to increase physical activity but also by their capacity to sustain independence and daily functioning among more vulnerable subgroups of older adults.

### 4.1 Limitations

Several limitations should be considered when interpreting these findings. First, AFCCQ scores were not assessed at the individual level within this cohort but were derived from a recent validation study and assigned based on neighbourhood-level mean values (15). Although neighbourhoods in Oldenburg are relatively homogeneous and linkage was performed using residential addresses, individual perceptions of age-friendliness may still vary within districts. Direct assessment of perceived age-friendliness at the individual level would allow more precise estimation of associations and reduce the risk of ecological misclassification. To account for clustering within city districts, GEEs with robust standard errors were applied. Initial multilevel random-intercept models did not converge, suggesting limited between-area variance within this sample. Second, the sample was drawn from participants of the SeFallED study, all of whom had recently presented to the emergency department following a fall (19). Although recruitment through this setting may increase sociodemographic heterogeneity, the experience of a recent fall may have influenced mobility behaviour, life-space mobility, or perceived functional capacity. While prior research suggests that physical activity levels may stabilise within months after a fall (50), residual effects cannot be excluded. Third, the cross-sectional design precludes causal inference. It remains unclear whether neighbourhood-level age-friendliness influences mobility and independence, or whether older adults with reduced mobility evaluate their environments differently. Longitudinal studies are needed to clarify temporal and bidirectional relationships. Finally, the relatively homogeneous urban context and generally high satisfaction with age-friendliness in Oldenburg may have limited variability in environmental exposure, reducing the ability to detect associations with physical activity. Replication in more diverse urban and rural settings is warranted.

## 5 Conclusion

In this sample of community-dwelling older adults after a fall, neighbourhood-level age-friendliness showed limited associations with active physical activity outcomes but was associated with sedentary behaviour, life-space mobility and functional independence in an inverse direction. These cross-sectional findings suggest that age-friendly environments may be particularly relevant for older adults with reduced mobility and functional limitations. However, longitudinal research is needed to clarify whether these associations reflect compensatory neighbourhood processes, reverse causality, or bidirectional relationships. Age-friendly city initiatives should, therefore, focus not only on promoting physical activity but also on supporting independence and everyday functioning in vulnerable older populations.

## Data Availability

The dataset used and analyzed during the current study will be made available by the corresponding author upon reasonable request. As public availability would compromise patient privacy, storing the data in a public repository is not possible. However, upon request and after approval by the local ethics board, the dataset may be made available. To request access to the data, please contact the corresponding author.
