# Supplementary material for: Neighbourhood-level age-friendliness in urban environments: associations with functional independence and physical activity in older adults after a fall
Source: Front Public Health. 2026 May 29;14:1839024. doi: 10.3389/fpubh.2026.1839024 (PMC13259899; doi:10.3389/fpubh.2026.1839024)
Supplement: Supplementary file 1 [file Table_1.docx]

Table S2: Associations of neighbourhood-level age-friendliness with physical activity

|  | **Predictor** | **B (SE)** | **95% CI** | **p-value** |
| --- | --- | --- | --- | --- |
| **Activity level (minutes)** | | | | |
| **Model 1** | AFCCQ total score | -3.82 (2.26) | -8.24 to 0.61 | .091 |
|  | Age (years) | -1.88 (0.45) | -2.76 to -1.00 | **<.001** |
|  | Sex (female vs male) | -20.64 (11.17) | -42.54 to 1.26 | .065 |
|  | Housing: non-barrier-free | 21.10 (8.43) | 4.58 to 37.63 | **.012** |
|  | Housing: barrier-free | 6.50 (11.22) | -15.49 to 28.49 | .562 |
|  | Living with partner | -25.35 (19.40) | -63.38 to 12.68 | .191 |
|  | Widowed | -43.03 (14.36) | -71.18 to -14.89 | **.003** |
| **Model 2** | Physical environment | -13.76 (32.30) | -77.06 to 49.54 | .670 |
|  | Age (years) | -1.62 (0.43) | -2.47 to -0.78 | **<.001** |
|  | Sex (female vs male) | -18.61 (11.59) | -41.32 to 4.10 | .108 |
|  | Housing: non-barrier-free | 20.33 (8.99) | 2.71 to 37.96 | **.024** |
|  | Housing: barrier-free | 5.83 (11.54) | -16.79 to 28.45 | .614 |
|  | Living with partner | -25.31 (19.32) | -63.19 to 12.56 | .190 |
|  | Widowed | -43.59 (14.08) | -71.18 to -16.00 | **.002** |
| **Model 3** | Social environment | -30.99 (13.81) | -58.04 to -3.93 | **.025** |
|  | Age (years) | -1.84 (0.43) | -2.68 to -1.00 | **<.001** |
|  | Sex (female vs male) | -20.02 (11.49) | -42.55 to 2.50 | .081 |
|  | Housing: non-barrier-free | 21.88 (8.53) | 5.16 to 38.59 | **.010** |
|  | Housing: barrier-free | 7.21 (11.76) | -15.85 to 30.26 | .540 |
|  | Living with partner | -24.91 (19.37) | -62.87 to 13.05 | .198 |
|  | Widowed | -43.68 (14.30) | -71.72 to -15.65 | **.002** |
| **Model 4** | Community-based services | -19.80 (17.50) | -54.09 to 14.50 | .258 |
|  | Age (years) | -1.84 (0.44) | -2.71 to -0.97 | **<.001** |
|  | Sex (female vs male) | -19.56 (11.01) | -41.13 to 2.01 | .075 |
|  | Housing: non-barrier-free | 21.46 (8.59) | 4.62 to 38.30 | **.012** |
|  | Housing: barrier-free | 6.48 (11.13) | -15.34 to 28.30 | .560 |
|  | Living with partner | -25.44 (19.45) | -63.56 to 12.69 | .191 |
|  | Widowed | -43.15 (14.43) | -71.44 to -14.86 | **.003** |
| **Energy expenditure (kcal)** | | | | |
| **Model 1** | AFCCQ total score | -215.97 (184.05) | -576.69 to 144.76 | .241 |
|  | Age (years) | -163.56 (43.35) | -248.52 to -78.60 | **<.001** |
|  | Sex (female vs male) | -2409.66 (743.68) | -3867.25 to -952.06 | **.001** |
|  | Housing: non-barrier-free | 1586.43 (622.67) | 366.01 to 2806.84 | **.011** |
|  | Housing: barrier-free | 443.76 (751.76) | -1029.67 to 1917.19 | .555 |
|  | Living with partner | -1649.79 (1448.51) | -4488.82 to 1189.25 | .255 |
|  | Widowed | -2983.67 (1110.41) | -5160.03 to -807.32 | **.007** |
| **Model 2** | Physical environment | -617.39 (2331.90) | -5187.84 to 3953.06 | .791 |
|  | Age (years) | -148.54 (40.98) | -228.85 to -68.23 | **<.001** |
|  | Sex (female vs male) | -2287.98 (767.30) | -3791.85 to -784.10 | **.003** |
|  | Housing: non-barrier-free | 1545.19 (645.39) | 280.25 to 2810.13 | **.017** |
|  | Housing: barrier-free | 409.54 (766.53) | -1092.83 to 1911.91 | .593 |
|  | Living with partner | -1648.05 (1443.19) | -4476.64 to 1180.55 | .253 |
|  | Widowed | -3019.08 (1096.29) | -5167.77 to -870.40 | **.006** |
| **Model 3** | Social environment | -2148.88 (1120.58) | -4345.18 to 47.42 | .055 |
|  | Age (years) | -164.45 (40.99) | -244.79 to -84.12 | **<.001** |
|  | Sex (female vs male) | -2399.92 (766.53) | -3902.30 to -897.55 | **.002** |
|  | Housing: non-barrier-free | 1647.43 (603.74) | 464.12 to 2830.74 | **.006** |
|  | Housing: barrier-free | 497.29 (764.99) | -1002.07 to 1996.64 | .516 |
|  | Living with partner | -1619.55 (1444.48) | -4450.67 to 1211.58 | .262 |
|  | Widowed | -3017.85 (1115.84) | -5204.86 to -830.84 | **.007** |
| **Model 4** | Community-based services | -1006.78 (1296.76) | -3548.38 to 1534.82 | .438 |
|  | Age (years) | -159.55 (43.32) | -244.45 to -74.66 | **<.001** |
|  | Sex (female vs male) | -2339.87 (735.49) | -3781.41 to -898.33 | **.001** |
|  | Housing: non-barrier-free | 1601.43 (625.91) | 374.67 to 2828.18 | **.011** |
|  | Housing: barrier-free | 440.93 (744.07) | -1017.42 to 1899.28 | .553 |
|  | Living with partner | -1654.21 (1451.26) | -4498.63 to 1190.22 | .254 |
|  | Widowed | -2994.59 (1114.06) | -5178.11 to -811.08 | **.007** |
| **Steps per day** | | | | |
| **Model 1** | AFCCQ total score | -109.53 (126.09) | -356.67 to 137.61 | .385 |
|  | Age (years) | -160.27 (36.34) | -231.50 to -89.03 | **<.001** |
|  | Sex (female vs male) | -466.21 (644.68) | -1729.77 to 797.34 | .470 |
|  | Housing: non-barrier-free | 457.09 (391.80) | -310.83 to 1225.02 | .243 |
|  | Housing: barrier-free | -308.11 (638.15) | -1558.86 to 942.65 | .629 |
|  | Living with partner | -586.14 (908.09) | -2365.97 to 1193.69 | .519 |
|  | Widowed | -694.81 (836.79) | -2334.89 to 945.27 | .406 |
| **Model 2** | Physical environment | -1779.81 (941.61) | -3625.34 to 65.72 | .059 |
|  | Age (years) | -157.01 (36.51) | -228.57 to -85.44 | **<.001** |
|  | Sex (female vs male) | -494.28 (638.44) | -1745.60 to 757.05 | .439 |
|  | Housing: non-barrier-free | 445.70 (377.73) | -294.64 to 1186.03 | .238 |
|  | Housing: barrier-free | -339.96 (589.86) | -1496.06 to 816.14 | .564 |
|  | Living with partner | -652.37 (871.69) | -2360.85 to 1056.11 | .454 |
|  | Widowed | -700.42 (792.87) | -2254.42 to 853.58 | .377 |
| **Model 3** | Social environment | -357.75 (683.25) | -1696.90 to 981.41 | .601 |
|  | Age (years) | -153.98 (36.51) | -225.53 to -82.43 | **<.001** |
|  | Sex (female vs male) | -428.64 (657.17) | -1716.67 to 859.38 | .514 |
|  | Housing: non-barrier-free | 458.11 (386.66) | -299.73 to 1215.95 | .236 |
|  | Housing: barrier-free | -302.60 (653.00) | -1582.45 to 977.25 | .643 |
|  | Living with partner | -588.59 (910.92) | -2373.95 to 1196.78 | .518 |
|  | Widowed | -724.08 (837.77) | -2366.09 to 917.93 | .387 |
| **Model 4** | Community-based services | -692.93 (976.96) | -2607.74 to 1221.88 | .478 |
|  | Age (years) | -159.99 (35.52) | -229.60 to -90.38 | **<.001** |
|  | Sex (female vs male) | -443.40 (649.82) | -1717.03 to 830.22 | .495 |
|  | Housing: non-barrier-free | 465.27 (406.66) | -331.77 to 1262.31 | .253 |
|  | Housing: barrier-free | -310.61 (648.20) | -1581.05 to 959.84 | .632 |
|  | Living with partner | -591.08 (912.34) | -2379.24 to 1197.08 | .517 |
|  | Widowed | -712.84 (838.97) | -2357.20 to 931.52 | .396 |
| **Sedentary time** | | | | |
| **Model 1** | AFCCQ total score | 9.87 (3.23) | 3.53 to 16.21 | **.002** |
|  | Age (years) | 2.38 (0.84) | 0.73 to 4.03 | **.005** |
|  | Sex (female vs male) | -21.40 (24.82) | -70.05 to 27.26 | .389 |
|  | Housing: non-barrier-free | -40.09 (25.72) | -90.51 to 10.32 | .119 |
|  | Housing: barrier-free | -26.41 (35.59) | -96.17 to 43.35 | .458 |
|  | Living with partner | 15.58 (14.08) | -12.02 to 43.18 | .268 |
|  | Widowed | 31.49 (30.53) | -28.35 to 91.33 | .302 |
| **Model 2** | Physical environment | 137.01 (26.26) | 85.53 to 188.49 | **<.001** |
|  | Age (years) | 2.00 (0.76) | 0.52 to 3.49 | **.008** |
|  | Sex (female vs male) | -19.89 (24.95) | -68.78 to 29.01 | .425 |
|  | Housing: non-barrier-free | -39.18 (24.75) | -87.69 to 9.34 | .114 |
|  | Housing: barrier-free | -23.93 (34.50) | -91.55 to 43.69 | .488 |
|  | Living with partner | 21.11 (14.55) | -7.40 to 49.62 | .147 |
|  | Widowed | 32.55 (31.45) | -29.09 to 94.19 | .301 |
| **Model 3** | Social environment | 34.17 (28.39) | -21.48 to 89.81 | .229 |
|  | Age (years) | 1.83 (0.68) | 0.50 to 3.16 | **.007** |
|  | Sex (female vs male) | -24.72 (24.11) | -71.97 to 22.53 | .305 |
|  | Housing: non-barrier-free | -40.21 (24.22) | -87.68 to 7.27 | .097 |
|  | Housing: barrier-free | -26.94 (35.56) | -96.64 to 42.75 | .449 |
|  | Living with partner | 15.64 (14.40) | -12.58 to 43.86 | .277 |
|  | Widowed | 34.02 (32.51) | -29.71 to 97.74 | .295 |
| **Model 4** | Community-based services | 49.98 (30.47) | -9.75 to 109.71 | .101 |
|  | Age (years) | 2.19 (0.79) | 0.65 to 3.73 | **.005** |
|  | Sex (female vs male) | -23.93 (24.67) | -72.28 to 24.42 | .332 |
|  | Housing: non-barrier-free | -40.63 (25.57) | -90.75 to 9.49 | .112 |
|  | Housing: barrier-free | -26.19 (35.67) | -96.10 to 43.72 | .463 |
|  | Living with partner | 16.53 (14.02) | -10.95 to 44.01 | .238 |
|  | Widowed | 33.66 (30.02) | -25.19 to 92.50 | .262 |

Table S3: Associations of neighbourhood-level age-friendliness with life-space mobility

|  | **Predictor** | **B (SE)** | **95% CI** | **p-value** |
| --- | --- | --- | --- | --- |
| **Life-space mobility** | | | | |
| **Model 1** | AFCCQ total score | -2.57 (1.39) | -5.29 to 0.15 | .064 |
|  | Age (years) | -1.51 (0.20) | -1.90 to -1.13 | **<.001** |
|  | Sex (female vs male) | -5.60 (4.57) | -14.57 to 3.36 | .220 |
|  | Housing: non-barrier-free | 0.76 (5.34) | -9.70 to 11.22 | .887 |
|  | Housing: barrier-free | -5.88 (6.95) | -19.49 to 7.74 | .398 |
|  | Living with partner | -7.85 (5.76) | -19.15 to 3.45 | .173 |
|  | Widowed | -10.28 (4.65) | -19.40 to -1.16 | **.027** |
| **Model 2** | Physical environment | -11.28 (17.69) | -45.96 to 23.39 | .524 |
|  | Age (years) | -1.33 (0.18) | -1.68 to -0.98 | **<.001** |
|  | Sex (female vs male) | -4.33 (4.95) | -14.02 to 5.36 | .381 |
|  | Housing: non-barrier-free | 0.05 (5.36) | -10.46 to 10.56 | .992 |
|  | Housing: barrier-free | -6.57 (7.30) | -20.87 to 7.74 | .368 |
|  | Living with partner | -7.71 (6.00) | -19.47 to 4.04 | .198 |
|  | Widowed | -10.95 (4.58) | -19.93 to -1.97 | **.017** |
| **Model 3** | Social environment | -16.10 (5.88) | -27.63 to -4.58 | **.006** |
|  | Age (years) | -1.44 (0.16) | -1.76 to -1.13 | **<.001** |
|  | Sex (female vs male) | -4.97 (4.56) | -13.90 to 3.97 | .276 |
|  | Housing: non-barrier-free | 1.04 (5.28) | -9.31 to 11.39 | .844 |
|  | Housing: barrier-free | -5.42 (7.45) | -20.03 to 9.18 | .467 |
|  | Living with partner | -7.63 (5.74) | -18.87 to 3.62 | .184 |
|  | Widowed | -10.85 (4.60) | -19.86 to -1.84 | **.018** |
| **Model 4** | Community-based services | -14.63 (9.79) | -33.81 to 4.55 | .135 |
|  | Age (years) | -1.51 (0.20) | -1.90 to -1.11 | **<.001** |
|  | Sex (female vs male) | -5.05 (4.48) | -13.84 to 3.74 | .261 |
|  | Housing: non-barrier-free | 0.88 (5.47) | -9.84 to 11.61 | .872 |
|  | Housing: barrier-free | -5.94 (7.04) | -19.73 to 7.85 | .399 |
|  | Living with partner | -7.84 (5.79) | -19.19 to 3.51 | .176 |
|  | Widowed | -10.15 (4.80) | -19.56 to -0.73 | **.035** |

Table S4: Associations of neighbourhood-level age-friendliness with instrumental activities of daily living

|  | **Predictor** | **B (SE)** | **95% CI** | **p-value** |
| --- | --- | --- | --- | --- |
| **Instrumental Activities of daily living** | | | | |
| **Model 1** | AFCCQ total score | -0.18 (0.03) | -0.24 to -0.11 | **<.001** |
|  | Age (years) | -0.08 (0.02) | -0.12 to -0.03 | **.001** |
|  | Sex (female vs male) | -0.13 (0.25) | -0.61 to 0.36 | .607 |
|  | Housing: non-barrier-free | -0.31 (0.27) | -0.83 to 0.21 | .245 |
|  | Housing: barrier-free | -0.70 (0.30) | -1.29 to -0.12 | **.018** |
|  | Living with partner | -0.39 (0.14) | -0.67 to -0.12 | **.005** |
|  | Widowed | -0.78 (0.19) | -1.15 to -0.41 | **<.001** |
| **Model 2** | Physical environment | -0.33 (0.86) | -2.01 to 1.35 | .702 |
|  | Age (years) | -0.06 (0.02) | -0.11 to -0.02 | **.006** |
|  | Sex (female vs male) | -0.02 (0.26) | -0.54 to 0.49 | .926 |
|  | Housing: non-barrier-free | -0.34 (0.31) | -0.95 to 0.26 | .266 |
|  | Housing: barrier-free | -0.74 (0.33) | -1.38 to -0.09 | **.025** |
|  | Living with partner | -0.39 (0.16) | -0.70 to -0.08 | **.014** |
|  | Widowed | -0.82 (0.20) | -1.22 to -0.42 | **<.001** |
| **Model 3** | Social environment | -1.03 (0.21) | -1.44 to -0.62 | **<.001** |
|  | Age (years) | -0.07 (0.02) | -0.12 to -0.03 | **.002** |
|  | Sex (female vs male) | -0.08 (0.25) | -0.57 to 0.40 | .738 |
|  | Housing: non-barrier-free | -0.30 (0.29) | -0.87 to 0.28 | .316 |
|  | Housing: barrier-free | -0.68 (0.35) | -1.36 to 0.00 | **.049** |
|  | Living with partner | -0.38 (0.14) | -0.64 to -0.11 | **.005** |
|  | Widowed | -0.81 (0.19) | -1.18 to -0.44 | **<.001** |
| **Model 4** | Community-based services | -1.29 (0.27) | -1.81 to -0.77 | **<.001** |
|  | Age (years) | -0.08 (0.02) | -0.12 to -0.03 | **<.001** |
|  | Sex (female vs male) | -0.11 (0.24) | -0.59 to 0.37 | .652 |
|  | Housing: non-barrier-free | -0.27 (0.28) | -0.81 to 0.27 | .329 |
|  | Housing: barrier-free | -0.69 (0.30) | -1.28 to -0.10 | **.023** |
|  | Living with partner | -0.40 (0.14) | -0.67 to -0.13 | **.004** |
|  | Widowed | -0.78 (0.18) | -1.13 to -0.42 | **<.001** |
